# GENERATING RESPONSES IMMUNE IN CELLULAR AND HUMORAL TREATMENT WITH EPITOPE SPIKE, EPITOPE ENVELOPE PROTEIN, AND EPITOPE MEMBRANE PROTEIN SARS-COV-2, HONEY, *SAUSSUREA LAPPA*, AND *NIGELLA SATIVA*

**DOI:** 10.21010/ajidv15i2S.3

**Published:** 2021-09-01

**Authors:** Sumarno Reto Prawiro, Meike Tiya Kusuma, Reyhan Amiruddin, Irma Nur Sukmawati, Yuyun Kusnaningrum, Jayshri Davi S Nadarajah, Khoirul Anam, Tri Yudani Mardining Raras, Sri Winarsih

**Affiliations:** 1 Department of Clinical Microbiology, Faculty of Medicine, University Brawijaya Malang Java Indonesia; 2 Magister Degree Program, Faculty of Medicine, University Brawijaya Malang Java Indonesia; 3 Clinical Microbiologist Degree Program, Faculty of Medicine, University Brawijaya Malang Java Indonesia; 4 Medical Doctor Degree Program, Faculty of Medicine, University Brawijaya Malang Java Indonesia; 5 Doctoral Degree Program, Faculty of Medicine, University Brawijaya Malang Java Indonesia; 6 Department Biochemistry Faculty of Medicine, University Brawijaya Malang Java Indonesia

**Keywords:** Respons immune, Honey, *Sausseria lappa*, *Nigella sativa*, COVID-19

## Abstract

**Background:**

Covid-19 has become pandemic in the World, including Indonesia. Our last study showed that HSF could serve as an immunomodulator. Using the exact search, we found that the most immuno-dominant SARS-COV2 epitope, namely A spike protein epitope, B envelope protein epitope, and C membrane protein epitope, we concise to be HF

**Materials and Methods:**

We used to post only control design study and mice as an animal model. The research divided mice into four groups, and the first group as control received PBS as a placebo. The second, three, and last four groups gave HF, HSN, and HFHSN (combine HF and HSN). All of the regiment enters the mouth with a special sonde to reach the gastrointestinal organ. We gave HF every week three times and HSN once a day. After administration regiments for a long three weeks, we sacrificed the mice. We evaluated cellular immune responses that are Th-2, Th-17, and NK cells. We check for humoral immune response, TGF-β,IL-17A, IL-4, IgG,IL-4, β-defensin, and s-IgA.

**Results:**

Highest profile cellular immunity HF, HSN, and HFHSN were NK cell, Th-2 and Th-17, and the last NK cell, respectively. After that which in humoral immunity, the domination response IgG and IL-4 were HF. But HSN and HFHSN dominated for s-IgA and β-defensin production. By using the study Bio-Informatica, we found HF.

**Conclusion:**

If the results of this study are continued to the clinical trial level, it is necessary to recommend additional markers such as CTL (s-IgA and β-defensin in lung tissue)and CPE assay.

## Introduction

Up to Jan 30, 2021, the World confirmed positive cases of Covid-19 as 101,561,219 and deaths of 2,196,944. Data for Indonesia shows a positive number of 1,051,795 with 29,518 deaths. One way to overcome this problem by scientists is to find a vaccine for COVID-19 (Wu, 2020).

Until now, some of the data from several countries have authorized nine types of vaccines. The vaccines are two RNA vaccines (the Pfizer - BioNTech vaccine and the Moderna vaccine). Then three conventional inactivated vaccines (BBIBP-CorV from Sinopharm, BBV152 from Bharat Biotech, and CoronaVac from Sinovac). Furthermore, three viral vector vaccines (Sputnik V from the Gamaleya Research Institute, the Oxford - AstraZeneca vaccine, and Ad5-nCoV from CanSino Biologics). Lastly, Russia’s Vector State Research Center of Virology developed a peptide vaccine (EpiVacCorona) that relies on chemically synthesized peptide antigens of SARS-CoV2 proteins conjugated to a carrier protein (Zimmer *et al.*, 2021).

The mucosal immune response most essential to kill virulent microbes that enter through the mucosa is s-IgA and β- defensin (Chairatana and Nolan, 2017). The pathogenesis of COVID-19, which mainly occurs due to the inhalation of air containing SARS-COV2. This virus enters the airway, then certain peptide parts in the spike attach to the ACE2 receptors on the airway epithelium’s surface (Gheblawi *et al.*, 2020). This biological mechanism is critical for the survival of SARS-COV2. Because after adhesion occurs, SARS-COV2 internalizes, then replicates, causing cell destruction (Yuki *et al.*, 2020). So that the ideal vaccine should be following pathogenesis, meaning that it can produce an adequate mucosal immune response in addition to an adaptive immune response.

The hope is that the presence of this mucosal immune response will inhibit adhesion and kill SARS-COV2. As a result, it is unable to internalize, and there is no damage to mucosal cells. The role of mucosal immunity is vital should the researchers think about giving the oral COVID-19 vaccine (Yuki *et al.*, 2020).

In our latest study, the results showed that the antibody epitope ATLGATLNRL-DFNVNNK (A-K) amino acid inhibited fluid into the intestinal mucosal cavity in mice infected with *S. flexneri*. We got this A-K amino acid epitope through bioinformatics tracking (Sumarno *et al.*, 2019). . The assumption of the dominance of the HF amino acids in the SARS-COV2 and A epitope will bind to ACE2.

As a carrier for the HF epitope, it is safe against stomach acid and enzymes in the digestive tract. We chose the ISCOM molecule. Our results show that ISCOM containing β-Glucan from *C. albicans* and AdhO36 *S. Typhi* has a potential effect on cellular and humoral immune response (Rachmawati *et al.*, 2020).

In believing Muslim citizens, many Indonesian people try to cured COVID-19 used HSN to refer to The Holy Quran An Nahl:69 (Ali, 2014) and Kitab Shohih (Khan, 1997). Many cases of COVID-19 use these preparations that do not require hospital treatment. Some reports are not well documented; many say a complete recovery.

Flavonoid compounds are found in honey, and these flavonoids have the potential to inhibit SARS-CoV2, 3-CLpro.

Subsequent studies have shown that flavonoids are potent inhibitors of the main protease 3CLpro (6LU7) SARS-CoV2 (Cheung *et al.*, 2019; Jo *et al.*, 2020; C. Wu *et al.*, 2020). Furthermore, based on the energy binding score in the insilico study, the syrigaresinol compound in *S. lappa* may have a lyses effect on the Coronavirus (Vincent *et al.*, 2020). Regarding the virucidal effects of Nigella sativa, including SARS-CoV2, it is still unclear. However, there are reports of the lysis of virus impact of Nigella sativa using immunomodulatory and anti-inflammatory effects. The thymoquinone content of Nigella sativa can affect signaling pathways, cellular immune responses, and humoral immune responses (Majdalawieh and Fayyad, 2015).

Based on the above background, our group conducted a study on giving the epitope FH, HSN, and FH and HSN’s combination on the immune response in mice. The results we can study together from our study by using the mice model.

## Materials and Methods

The present study was conducted by using the post-only design control method according to the following stages:

## ISCOM preparation

The ISCOM formulation uses 5 mg Quail A Saponin + 10 mg / ml epitope FH (in 0.2 M PBS pH 6). Next we dissolved 100 µL of (B) 1% cholesterol + 1% Phosphatidylcholine solution in 20% Mega 10 egg yolk lectins. Homogenization using a magnetic stirrer at room temperature. Next, dialysis in 0.2 M PBS pH 7.4 at room temperature for 2-3 hours. Proceeded on overnight dialysis but at 4°C. Continue the ISCOM preparation by centrifugation 10,000 g x 5 ’. The results of the supernatant centrifugation were 300 µl + 25% sucrose (MW = 342.3 g / mol) in 0.2 PBS pH 7.4 (1: 1). Next, we performed ultracentrifugation 257,000 x g 2 hours.

Researchers took the precipitate fraction with a needle (measuring the protein concentration measured by nanodrop by dissolving in PBS 2.5 ml pH 7.4). Finally, as confirmation, we made observations on the Transmission Electron Microscope (TEM) (Mowat *et al.*, 2001).

## HSN preparation

To calculate the dose for the animal model, we do as follow, and we divided the HSN dosage by the number 5000 ml (human blood volume) / 5 ml (mouse blood volume) to find the dose according to the mice. So we found a Honey dose of 5/1000 ml

= 0.005 ml, *Sausseria lappa*/1000 ml = 0.05 ml, and *Nigella sativa* 2/1000 ml = 0.002 ml for each administration. Immunization to generate an immune response, we give it orally. Treatment one was controlled with PBS; we gave treatment two immunization with FH, treatment three with HSN, and the last treatment with HFHSN.

We subjected each mouse to immunization containing an epitope of 25µg in volume per 100µl. We gave immunizations three times every week and isolated the sample one week after the last vaccination.

## Flow cytometry examination

We measured the number of Th-17, Th-2, and NK cells using flow cytometry. The Th-17 examination used anti-mouse CD4 FITC antibody and IL-17 anti-mouse PE by looking at the expression of CD4 and IL-17A. After that, the Th-2 marker used the anti-mouse CD4 FITC antibody and the IL-4 anti-mouse PerCP antibody. Simultaneously, the NK cells used the CD-161 anti-mouse APC antibody marker (Muhyi *et al.*, 2015).

## ELISA examination

We measured IL-17, IL-4, TGF-β, IgG levels from serum samples while s-IgA and β-defensin from mucus samples by ELISA method using ELISA kit. The procedure our carried following the system in the ELISA kit. IL-17, IL-4, TGF-β using the ELISA kit from BioLegend (San Diego, CA, USA). We cut the intestine at 10 cm in length. Mucous collected by scraping from the intestine after removing the entrail and pooled mucous containing rich s-IgA and β-defensin, we suspended with a 1: 1 PBS ratio. After then the mucous was centrifuged at 6000 rpm for 30 min at 4^o^ C temperature. Then we stored the supernatant at 4^o^ C. We used An ELISA kit from the Bioassay Technology Laboratory (Shanghai. China) to check for s-IgA and β-defensin.

## Ethical Approval

The Ethics Committee of the Faculty of Medicine, University Brawijaya Malang, Indonesia, approved this study (Approval No 044-KEP-UB-2020).

## Data analysis

The reachers expressed the results obtained as Mean ± SD then compared using an unpaired two-tailed Student’s T-test

## Results

**Figure 1:**
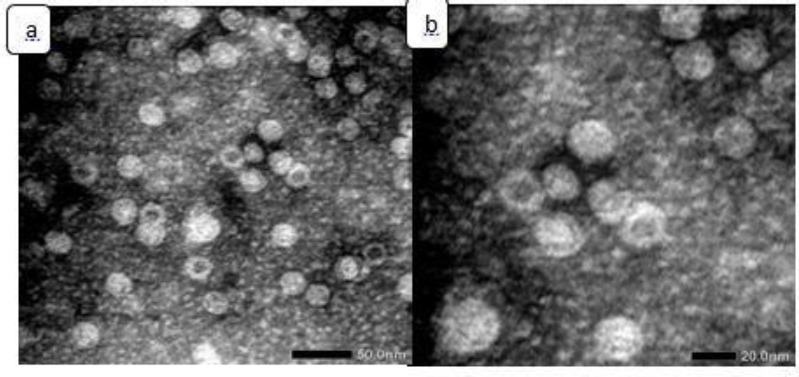
Results of observations of fusion SAR-COV2 with ISCOM on the Transmission Electron Microscope (TEM).

We used adjuvants ISCOM to carry epitopes in immunization in mice. The ISCOM can have antigens, and we could see them by using a TEM microscope. Examination results using a TEM microscope against epitope antigens with ISCOM to know the effectiveness of ISCOM in carrying antigens (Fig 1).

Morphology ISCOM, which carried Epitope SARS-COV2. a) ISCOM + epitope morphology with a magnification of 50 nm; b) ISCOM + epitope morphology with a magnification of 20 nm.

Our observations using a TEM microscope show that ISCOM fuses with the epitope. Fig. 1 shows a picture of a caged ball. The white ball signifies that the epitope is in ISCOM, while the empty ball indicates that the epitope has not entered.

**Figure 2:**
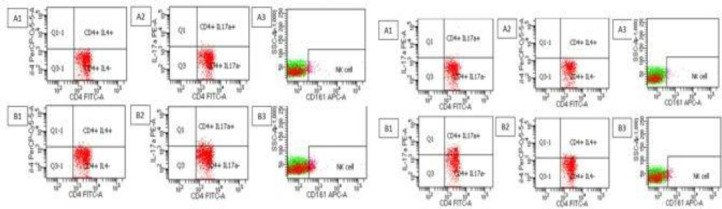
Th-2, Th-17, and NK cell sample treatment (HF) amino acids using flow cytometer.

The image to the side shows the treatment (Control, HF, HSN, HFHSN). The image below shows the parameters (Th2, Th17, NK cell). Based on Fig 2. (from one of the Flowcytometry results), all treatments and parameters differ from the control group. We can see in the right-hand quadrant the number of Th2 cells. Th2 cells are marker-carrying T cells, whereas we use the CD4 + and IL4 markers. A significant increase in Th2 cells occurred in HSN treatment. To enhance Th-17 cells, we can see a right-hand quadrant image showing CD4 + and IL-17 + expression. The increase in Th17 cells also occurred significantly in HSN treatment. Furthermore, to increase NK cells’ number in the image we selected, the gate is a population that expresses NK cells. The number of NK cells increased significantly in the HFHSN treatment.

**Figure 3:**
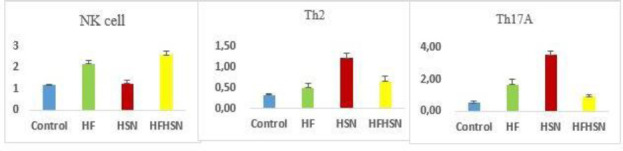
Response Immune cellular treatment, with HF, HSN, and combination. HF, three kinds of epitope spike SARS- COV2, HSN, Honey, S. coctus, and N. sativa, and HFHSN combines HF with HSN.

**Figure 4:**
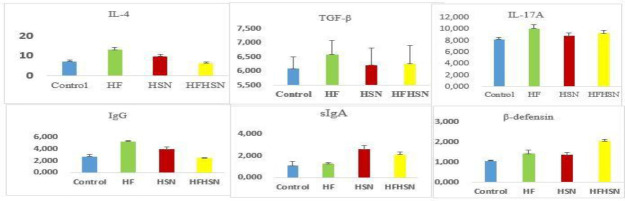
Results of cellular immune response due to exposure with HF, HSN, and HFHSN We gave three kinds of treatment HF (3 types of SARS-COV2 epitope), HSN (Honey, *S. lappa*, and *N. sativa*), and HFHSN

The treatment of the SARS-COV2 spike protein epitope increases cellular immune response, namely Th-2, Th-17, and NK cells, and an increase in the humoral immune response of TGF-β, sIgA, IL-4, β-defensin, IL-17, and IgG. The results of the rise in the cellular immune response we shown in Fig 2 and Fig 3. Fig 4 and Table 1 show the results of the increase in the humoral immune response.

**Table 1:** Response Immune cellular treatment with HF, HSN, and HFHSN

Cellular Immunity	Treatment	Mean±SD	p	Sig.
±				

Th-2	Control	0.32±0.04	0.28 - 0.36	a

	HF	0.50±0.10	0.40 - 0.60	b

	HSN	1.22±0.13	1.09 - 1.35	d

	HFHSN	0.66	0.53 - 0.79	c

Th-17A	Control	0.50±0.12	0.38 - 0.62	a

	HF	1.66 0.32	1.34 - 1.98	c

	HSN	3.54±0.25	3.29 - 3.79	d

	HFHSN	0.92±0.08	0.84 - 1.00	b

NK cell	Control	1.16±0.05	1.11 - 1.21	a

	HF	2.16±0.17	1.99 - 2.33	b

	HSN	1.24±0.15	1.09 - 1.39	a

	HFHSN	2.58±0.19	2.39 - 2.77	c

HF: 3 types epitopes SAR-COV2, HSN: Honey, *S. lappa*, and *N. sativa*, and HFHSN: combination HF and HNS

HF: 3 types of epitopes SAR-COV2, HSN: Honey, *S. lappa, N. sativa*, and HFHSN: combination HF and HSN.

Table 1 and Fig. 3 show significant or insignificant statistical test results (p <0.5). The cellular immune response due to HSN exposure produced the highest Th-2 cells. The same products we found in population Th-17. Meanwhile, exposure to HFHSN resulted in the lowest NK cell populations, followed by FH and HSN.

HF: 3 types of epitopes SAR-COV-2, HSN: Honey, *S. coctus, N. sativa*, and plHFHSN: combination HF and HNS.

(the combination of HF with HSN). Our exposure to 4 groups of mice yields Table 2 and Fig. 4. Table 2 and Fig. 4 are attractive to discuss the polarization of outcomes important for humoral immune response. These humoral immune responses are β-defensin, s- IgA, and IgG. Researchers saw the highest amount of β-defensin due to exposure to HFHSN. As for the results of HSN exposure, we saw the highest increase in s-IgA. At the same time, HF showed the highest IgG humoral response. Researchers also need to discuss other exciting markers, namely IL-4, IL-17A, and TGF-β.

**Table 2:** Results of humoral immune response due to exposure to HF, HSN, and the combination of HF and HFHSN

IL-4					TGF-β			
T	**C**	**HF**	**HSN**	**HFHSN**	**C**	**HF**	**HSN**	**HFHSN**
M	7.103	13.134	9.785	6.257	6.09	6.59	6.21	6.26
	±0.81	±1.157	±1.07	±0.574	±0.417	±0.48	±0.62	±0.65
p	ab	c	d	a	a	b	ab	c

β-defensin					IL17A			

T	**C**	**HF**	**HSN**	**HFHSN**	**C**	**HF**	**HSN**	**HFHSN**

M	1.05	1.41	1.36	2.05	8.22	10.09	8.82	9.22
	0.05	0.17	0.12	0.07	0.34	0.69	0.52	0.52
	±	±	±	±	±	±	±	±
p	a	b	b	c	a	b	a	ab

s-IgA					IgG			
T	**C**	**HF**	**HSN**	**HFHSN**	**C**	**HF**	**HSN**	**HFHSN**
M	1.10	1.25	2.584	2.09	2.72	5.26	3.94	2.45
	±0.39	±0.12	0.39	0.23	0.31	0.15	0.41	0.14
			±	±	±	±	±	±
p	a	a	b	c	a	b	c	a

T: Treatment, M: Mean, p: significance, HF: 3 types epitopes SAR-COV-2, HSN: Honey, *S. coctus*, and *N. sativa*, and HFHSN: combination HF and HNS

## Discussion

Humans who breathe the air that contains SARS-COV2, the virus will start the pathogenesis process of COVID19 (Chairatana and Nolan, 2017). Furthermore, SARS-COV-2 will attach to the cylindrical epithelial surface as the lining cells of the airway surface. The ability of the virus to attach to epithelial cells is due to the compatibility of its receptors. We found the receptor on the lower and upper airways (Gheblawi *et al.*, 2020). The study results have clarified that this receptor is an angiotensin-converting enzyme 2 (ACE2). Spike comprises two functional subunits; the S1 subunit is responsible for binding to the host cell receptor, and the S2 subunit is for the fusion of the viral and cellular membranes (Yan *et al*. 2020). The ligand of ACE2 no one has searched yet molecularly; researchers have found that its location is part of the spike of SARS-COV-2. ACE2 for the ligand in SARS- COV-2 attaches to ACE2. The following process is the virus internalizing it into the epithelial cells where it is attached. Finally, the virus replicates rapidly, damaging epithelial cells from the upper and lower airways where ACE2 is present. We understand that this attachment process is essential for the survival of this SARS-COV-2 virus (Yan *et al*. 2020).

To prevent this sticking process on the body’s mucosal layer, we try to repel or kill any pathogenic microbes that pass through the mucosa. As a result, we found two kinds of immune response results in our mucosa, namely β-defensin and s-IgA (Chairatana and Nolan, 2017).

The microbicidal/virucidal β-defensin mechanism is virucidal directly lysis. β-defensin has a positive electron charge while the microbe has a negative charge. The emergence of this positive and negative charge bond will lyse the microbes (Borsutzky *et al.*, 2004; Wilson *et al.*, 2016). As in Table 2 and Fig. 4, we hope that combining the three types of epitopes (HF) with honey, *S.coctus*, and *N. sativa* (HSN) will trigger the production of β-defensin. Our results showed that in the four treatment groups, it turned out that HFHSN produced the highest β-defensin, and in the control group, the lowest. Meanwhile, the mechanism of s-IgA lyses microbes/viruses indirectly. The microbial/viral death process requires the help of dendritic cells in the submucosa to protrude into the mucous fluid. This bulge will catch s-IgA, which opzonized/binds to microbes/viruses (phagocytosis process) (Blaschitz and Raffatellu, 2010). In Table 2 and Fig. 4, it turns out that the s-IgA profile (which is present in the intestinal mucosa) is different from the β-defensin profile (Cerutti, 2008).

Some studies reported that SARS-COV2 could enter the gastrointestinal and show the gastroenteritis symptom. Some patients may complain of abdominal pain, vomiting, and diarrhea, instead of respiratory symptoms (Pan *et al.*, 2020). Our study got s-IgA from the intestine and jejunum. This finding is exciting and maybe could protect SARS-COV-2, which enters the gastrointestinal tract.

Our study lost a second of the opportunity to check s-IgA in the mucous respiratory tract, and the first is CTL, as we have mentioned. s-IgA in the mucous respiratory tract should elevate due to the homing plasma cell from the gut go to the respiratory tract (Pakkanen *et al.*, 2010). So subsequent study, we have to check s-IgA and β-defensin. How is the Th-17 precursor of β-defensin homing from the gastrointestinal tract to the respiratory tract? We should elaborate. Our recent study shows that Th-17 in lien elevated if vaccine candidate Cholera gave per oral (Prawiro *et al.*, 2020).

We compared the HSN results. HF, HFHSN, and control treatments showed higher HSN values. We did not check for specific s-IgA for the three types of epitopes we used. So that our test results are the whole s-IgA, we elaborate on this problem in future research. From the results of this study, we temporarily conclude that HSF causes an increase in mucosal immune response, which has an immunoadjuvant role.

By presenting the results in Table 2 and Fig. 4, we also know the IgG profile. The IgG that we examined was in the serum fluid, and in Fig. 4, IgG was the highest in the HF, HSN, HFHSN, and control groups. The role of IgG such as s-IgA to lyse microbes

requires the help of effector cells, namely macrophages, which will phagocyte microbes that bind to IgG/opzonazation (Thau *et al.*, 2021). Another effector cell is the NK cell, which can bind IgG. Fab, a part of IgG, and IgG bound to NK cells, can bind to microbes attached to individual cells (Hashimoto *et al.*, 1983).

Whether the serum IgG and s-IgA produced are protective or not, we should do the (CPE) test. We cannot do this CPE yet. If possible, our study will be complete. The Fab fraction binding from antibodies attached to NK cells will lead to lysis of these NK cells, microbes, and specific cells. This situation is ADCC (antibody-dependent cellular cytotoxicity) (Von Holle and Moody, 2019).

In Fig. 3, we observed a statistically different (p≤0.05%) NK cell production between the HF and HFHSN groups than the control and HSN groups. The producer of humoral antibody (IgG) is B cell due to the role of Th-2, which produces IL-4 as an activator of B cells to produce antibodies. In Fig. 3, Th-2, HSN, and HFHSN significantly led to the proliferation of Th-2 groups.

The complement cascade reaction with the role of IgG can lyse microbes. Complement in the form of a liquid that is in the serum is a peptide. Complement requires a cascade reaction where the result is direct lysis of the microbes using a membrane attack complex (MAC). IgG will activate complement via the classical pathway, and we in this study did not evaluate the role of complement that can lyse microbes (Stoermer and Morrison, 2011).

Another cell, namely CTL (cytotoxic T lymphocytes), has the function of independently lysing microbes. This CTL will bind to MHC1 molecules on the surface of specific cells. The entry of microbes, especially viruses, into specific cells will express MHC1 molecules, and what will happen is identical to NK cells, which causes ADCC, but CTL does not require antibodies. In this study, we also missed an opportunity to evaluate the role of HF, HSN, and HFHSN on the CTL profile. By conducting additional studies on the part of complement, CTL, and CPE neutralization tests in this study, it will be possible to get a more comprehensive conclusion on whether HF and HSN are effective oral vaccine candidates (Uzhachenko and Shanker, 2019).

Vincent in 2020 carried out molecular docking to identify a potent inhibitor of the main protease 3CLpro (6LU7) SARS- CoV2. Bioinformatics studies using various medicinal plants recommended as drugs for SARS-CoV2. One of them is *S. lappa*, and syrigaresinol in *S. lappa* shows excellent antiviral properties compared to synthetic drugs. Meanwhile, based on the energy binding score, it turns out that this syrigaresinol compound can be tested whether it can fight the Coronavirus. So that for the future, it can be expected to develop effective antiviral drugs (Vincent *et al.*, 2020). Several studies also explained that flavonoid compounds present in certain plants showed good docking affinity against 3CLpro SARS-CoV2 (Jo *et al.*, 2020). We also found reports regarding the mechanism of the immunomodulatory and anti-inflammatory effects of *N. sativa*. The thymoquinone content of *Nigella sativa* can affect signaling pathways, cellular immune responses, and humoral immune responses (Majdalawieh and Fayyad, 2015)

Cholerae vaccine used nowadays, such as lyophilized CVD 103-HgR, Dukoral, has replaced the ChoTyPa vaccine used more than 60 years ago. The vaccine component for Cholerae, Thyfoid fever, Parathyfoid has an inactivated bacterial (Stellfeld, 2004). Because ChoTyPa’s protectivity rate was low, and WHO stopped it. Now for the cholera vaccine using a Ducoral vaccine given per oral, generating a mucosal immune response. To develop this vaccine, the inventor refers to the pathogenesis process in which *V. cholerae* attaches to the jejunum and ileum receptors, and the Dukoral vaccine contains adhesion molecules. For the COVID-19 vaccine, there are three conventional inactivated vaccines (BBIBP-CorV from Sinopharm, BBV152 from Bharat Biotech, and CoronaVac from Sinovac), and we will see the recovery rate in the future (Brandtzaeg, 2013).

When we compare our vaccine candidates with nine other vaccines that have been and will be circulating, there are advantages. The advantage is that we need a relatively short time of four months and cheaper costs, and the way and route of giving are easy. If a mutation, especially in the ligand in the SARS-COV2 spike, a virulence factor, or another new virulence factor appears, we quickly add or replace the epitope. Furthermore, we can give to all age groups. But it has limitations and weaknesses, namely its adequate short time.

## Conclusion

This study indicates that HF, HSN, and HFHSN affect the increase in adaptive cellular immunity, namely Th-2, Th-17, and NK cells. Likewise, the role of increasing humoral immunity, namely IgG. Besides, HF, HSN, and HFHSN can boost the immunity of s-IgA and β-defensin in the intestinal mucosa. The enhancement of markers of immunity of Th-2, Th-17, NK cells, s-IgA, and β- defensin from the immune response in this study is significant to kill microbes present in the mucosa, bloodstream, and organs. Thus, the role of HSN in our analysis of the HF immune response is as an immunomodulator.

If the results of this study are continued to the clinical trial level, it is necessary to recommend additional other markers. The markers that should not be overlooked in further studies are CTL (s-IgA and β-defensin in lung tissue) and CPE assay.

**Conflict of Interest Declaration:** The authors declare no conflict of interest associated with this study.

**Abbreviations:**
HSF: Honey, *Sausseria lappa*, and *Nigella sativa*
A: FLVLLPLVSSQCVNFLVLLPLVSSQCVNL epitope
B: VNSVLLFLAFVVFLLVTLASS epitope
C: NYACLIFVWPVT epitope
HF: A+B+C.
